# Synaptic dynamics and neuronal network connectivity are reflected in the distribution of times in Up states

**DOI:** 10.3389/fncom.2015.00096

**Published:** 2015-07-29

**Authors:** Khanh Dao Duc, Pierre Parutto, Xiaowei Chen, Jérôme Epsztein, Arthur Konnerth, David Holcman

**Affiliations:** ^1^IBENS, Ecole Normale Supérieure, Applied Mathematics and Computational BiologyParis, France; ^2^Institute of Neuroscience, Technische Universität MünchenMunchen, Germany; ^3^Institut de Neurobiologie de la Méditerranée-INSERM U901Marseille, France

**Keywords:** modeling, inverse problem, synaptic depression, Up states, non-poissonnian distribution, first passage times, mean-field model, neuronal networks

## Abstract

The dynamics of neuronal networks connected by synaptic dynamics can sustain long periods of depolarization that can last for hundreds of milliseconds such as Up states recorded during sleep or anesthesia. Yet the underlying mechanism driving these periods remain unclear. We show here within a mean-field model that the residence time of the neuronal membrane potential in cortical Up states does not follow a Poissonian law, but presents several peaks. Furthermore, the present modeling approach allows extracting some information about the neuronal network connectivity from the time distribution histogram. Based on a synaptic-depression model, we find that these peaks, that can be observed in histograms of patch-clamp recordings are not artifacts of electrophysiological measurements, but rather are an inherent property of the network dynamics. Analysis of the equations reveals a stable focus located close to the unstable limit cycle, delimiting a region that defines the Up state. The model further shows that the peaks observed in the Up state time distribution are due to winding around the focus before escaping from the basin of attraction. Finally, we use *in vivo* recordings of intracellular membrane potential and we recover from the peak distribution, some information about the network connectivity. We conclude that it is possible to recover the network connectivity from the distribution of times that the neuronal membrane voltage spends in Up states.

## Significant statement

Neuronal networks generate complex patterns of activity that depend on functional synapses. Synaptic dynamics generates network dynamics characterized by long periods of depolarization called Up states, that can last for hundreds of milliseconds. Yet understanding the network connectivity and the mechanisms underlying these periods remain unclear. Using a mean-field model, we find that the distribution of times in Up states does not follow a Poissonian law, but presents several unexplained peaks. These peaks are not artifacts of electrophysiological measurements, but result from the inherent dynamics properties of the network. Moreover, the present method allows resolving a reverse problem: how to extract the neuronal network connectivity from the distribution of times in a time series (Up states). We conclude that the distribution of times in Up states is due to synaptic dynamics in a neuronal network with sufficient connections and the position of peaks in the time distribution characterizes the degree of functional network connectivity, usually difficult to estimate.

## 1. Introduction

The cerebral cortex is continuously active even in the absence of external stimuli, showing patterns of activation that resemble the ones generated by direct stimulations (Kenet et al., 2003; Chen et al., 2013). Recurrent patterns have also been found in neuronal ensembles (Cossart et al., 2003). Yet the origin of this recurrent activity based on network properties remains unexplained. Several computational studies have addressed successfully the role of noise in generating oscillations in recurrent networks (Verechtchaguina et al., 2006; Nesse et al., 2008). The spontaneous activity of the membrane potential of connected neurons has further revealed that it can switch between an Up and a Down state (Lampl et al., 1999; Anderson et al., 2000; Chen et al., 2013).

This Up and Down state phenomena has been modeled for excitatory network by synaptic-depression mean-field equations (Tsodyks et al., 1998; Torres et al., 2002; Holcman and Tsodyks, 2006), where early simulations suggested that the residence time of the membrane potential in the cortical Up state does not follow a Poissonian law, but presents several unexplained peaks. Using a mean field model, we show here that these peaks are neither artifacts of numerical simulations nor electrophysiological measurements, but are rather an inherent property of the underlying dynamics.

Using excitatory synaptic transmission only, we shall see that the model used in this manuscript reproduces experimental settings, where inhibition is shut down in picrotoxin condition. By analyzing the times in Up states, we will show that their distribution presents oscillation peaks, allowing a novel characterization of time series recording of Up and Down states. We show in Supplementary Information that adding inhibition affects but do not change the nature of the oscillation peaks. Note that these peaks were never reported before and are completely different from the phenomenon of stochastic amplification of the voltage in Up state, where fluctuations of the mean rate in the Up states shows a single peak in the power spectrum (Hidalgo et al., 2012).

We present here a model and a method to analyze the mean properties of neuronal networks and in particular to recover the degree of connectivity from the distribution of times in depolarized states. We apply our analysis to *in vivo* recordings of intracellular membrane potential. The first part of our approach consists in studying a mean-field model and we find that the Up states are characterized by a basin of attraction where the stable focus is located near the unstable limit cycle. For a specific range of the noise amplitude, we demonstrate that the distribution of times in Up states, which is the survival probability of the Fokker-Planck equation has periodic decreasing peaks. The period of this oscillatory resonance is surprisingly exactly the imaginary part of the eigenvalue of the jacobian of the vector field at the focus, in agreement with asymptotic computations, obtained for generic dynamical systems (Dao Duc et al., 2014). We conclude that neuronal network driven by synaptic depression operates in a regime of parameters that leads to non-Poissonian residence time in the Up state. Finally, recovering the network connectivity from the distribution of times of the mean neuronal membrane voltage or in general from the distribution of firing rates, is a general concept that should be applied to neuronal networks of various sizes.

## 2. Materials and methods

### 2.1. Mean-field equations for synaptic-depression

A neuronal network connected by excitatory connections is described here by its mean firing rate averaged over the neural population. When the neuronal network is mostly connected with depressing synapses, the state of a synapse is represented by the depression parameter μ (Tsodyks and Markram, 1997). The mean neuronal activity follows the stochastic system (Bart et al., 2005; Holcman and Tsodyks, 2006)

(1) $$\begin{array}{l} \tau \dot{V}=-V+JU\mu R(V)+\sqrt{\tau }\sigma \dot{\omega }\\ \dot{\mu }=\frac{1-\mu }{t_{r}}-U\mu R(V), \end{array}$$

where *V* is an average voltage (measured in mV with a base line at 0 mV), *J* is the average synaptic strength in the network, *U* and *t*_*r*_ are utilization parameters and recovery time constants of the synaptic depression respectively (Tsodyks and Markram, 1997; Bart et al., 2005). We use a δ-correlated white noise $\overset{{}^{\circ}}{\omega }$ of mean zero and variance one (Schuss, 2010). The first term on the right-hand side of the first equation of Equation (fdt) accounts for the intrinsic biophysical decay to equilibrium. The second term represents the synaptic input, which is scaled by the synaptic depression parameter μ. The third one summarizes all uncorrelated sources of noise with amplitude σ. *R*(*V*) is the average firing rate (in Hz) approximated by a threshold-linear voltage dependence function

(2) $$R(V)=\{\begin{array}{ll} \alpha (V-T) & \text{ if }V>T\\ 0 & \text{ else}. \end{array}$$

where *T* > 0 is a threshold and α = 1*Hz*∕*mV* is a conversion factor. In general, the function *R*(*V*) is a sigmoid because it should account for the saturation of the neuronal firing rate. However, due to the synaptic-depression, the dynamics never reaches the saturation regime and we can thus use the function *R*(*V*) defined above. The second equation in fdt describes the activity-dependent synaptic depression according to the classical phenomenological model (Tsodyks and Markram, 1997): briefly, every incoming spike leads to an abrupt decrease in the instantaneous synaptic efficacy, measured by a utilization factor *U*, due to depletion of neurotransmitters. Between spikes, the synaptic efficacy recovers to its original state (μ = 1) with a time constant *t*_*r*_ (see parameters in Table 1). Entering into an Up state corresponds to an escape event in the region between the separatrices and the unstable limit cycle. It is known that the dynamics shows a fast rotation (population spike) around the unstable limit cycle and a small noise fluctuation can produce the impulsion necessary to push a trajectory into an Up state (Holcman and Tsodyks, 2006). This model has been used to describe excitatory neuronal network.

**Table 1:** **Simulation parameters (Holcman and Tsodyks, 2006)**.

**τ**	***U***	***J***	**σ**	***T***	***t*_*r*_**
0.05 s	0.5	12.6 mV/Hz	2.2 mV	2 mV	0.8 s

### 2.2. Experimental protocols

#### 2.2.1. *In vivo* whole-cell recording in the barrel cortex

Experimental procedures were performed in accordance with the recommendations of the French Institut National de la Santé et de la Recherche Médicale animal care and use committee and the European Council Directives (2010/63/UE). A male wistar rat (P27/87 g) was anesthetized briefly with isoflurane followed by urethane (1 g/kg body weight, i.p.) plus additional doses of a mixture of ketamine (50 mg/kg) and xylazine (2.5 mg/kg) as needed then head-fixed into a stereotaxic frame. A local analgesic (lidocane) was injected under the skin before the first incision. The skull was exposed and a small craniotomy was performed over the somatosensory cortex (2.5 mm posterior and 5.5 mm lateral to the bregma). Blind *in vivo* whole-cell recordings were obtained using previously described procedures (Margrie et al., 2002). Borosilicate glass patch pipettes (resistance 5–7 M?) were filled with an intracellular solution containing (in mM): 130 KMeSO4, 5 KCl, 5 NaCl, 10 HEPES-K, 2.5 MgATP, 0.3 NaGTP, 0.2 EGTA and 0.1 % of biocytin (pH adjusted to 7.2) and advanced into the brain perpendicular to the surface of the cortex. The cell membrane potential was recorded in current clamp mode without any current injection. The signal was amplified using an Axoclamp-2B amplifier (Molecular devices, Sunnyvale, California), low-pass filtered online at 3 kHz and digitized with a digidata 1440A (Molecular devices) at 20 kHz.

#### 2.2.2. *In vivo* whole-cell patch-clamp recording of layer 2/3 neurons in the auditory cortex

Whole-cell patch-clamp recordings of layer 2/3 neurons in the auditory cortex of C57BL/6 mice (30–50 postnatal days old) were obtained using the procedure as described previously (Kitamura et al., 2008; Jia et al., 2010; Chen et al., 2011). Briefly, the mouse was placed onto a warming plate (37.5–38°C) and anesthetized with 0.8–1.2% isoflurane in pure O2. The recording chamber was perfused with normal Ringer's solution containing (in mM): 125 NaCl, 4.5 KCl, 26 NaHCO3, 1.25 NaH2PO4, 2 CaCl2, 1 MgCl2, and 20 glucose , pH 7.4, when bubbled with 95% O2 and 5% CO2. The body temperature of the mouse was maintained in the range of 36.5–37.5°C throughout the recording period. Somatic current-clamp recordings were performed with an EPC-10 amplifier (USB Quadro Amplifier, HEKA Elektronik, Lambrecht/Pfalz, Germany) under two-photon imaging guidance. Borosilicate glass pipettes with open tip resistance of 5–7 MΩ were filled with an intracellular solution containing: 112 mM K-gluconate, 8 mM KCl, 10 mM HEPES, 4 mM Mg-ATP, 0.3 mM Na2GTP, 10 mM Na-Phosphocreatine, titrated to pH 7.20–7.25. The pipette series resistance was continuously measured and neurons were rejected for data analyses if the resistance was higher than 30 MΩ. Electrophysiological data were filtered at 10 kHz and sampled at 20 kHz using Patchmaster software (HEKA, Lambrecht, Germany). Two-photon imaging was performed with a custom-built video-rate two-photon microscope based on a resonant scanner (Leybaert et al., 2005) and a mode-locked femtosecond pulse laser, operating at 710–920 nm wavelength (MaiTai, Spectra Physics, Mountain View, CA). The scanner was mounted on an upright microscope (BX61WI, Olympus, Tokyo, Japan) equipped with a 40/0.80 water-immersion objective (Nikon, Japan).

### 2.3. Fitting procedure

We fitted the distribution of time in the Up state using the following fitting procedure:

1. We first fitted the first exponential *Ae*^−λ_0_*t*^ using the Matlab fitting toolbox using the constraint: 0.9λ^~^_0_ ≤ λ_0_ ≤ 1.1λ^~^_0_.λ^~^_0_ = 1/*MFPT*, where the MFPT is computed by averaging the time in the Up state.
2. We fitted the second exponential *Be*^−λ_1_*t*^ by subtracting the first one from the data and by considering the convex hull of the resulting curve. λ_1_ was manually adjusted maintaining the condition that it is larger than λ_0_.
3. We fitted the periodic part cos(ω*t* + ϕ) by manually finding the period *T* between two consecutive peaks on the histogram with $\omega =\frac{2\pi }{T}$.

The Up state histogram is obtained from electrophysiological recordings lasting 300 s of neurons located in the barrel cortex. The data were down-sampled by 100 Hz and the minimal value was subtracted so that now it is equal to zero. We annotate the Up states defined by a starting spike, followed by a period of stable depolarized membrane potential and the end is usually a single exponential decay. From the Up and Down states classification, we extracted the histogram of the Up states duration. It is composed of 325 Up states, that we bin into epochs of 0.0293 s (70 bins for 2.05 s). The histogram is presented in **Figure 8A**.

We also analyzed the histogram of Up states, recorded in the auditory cortex from 5 different electrophysiological traces, each lasting 32 s, obtained *in vivo* from a neuron of the L2 cerebral cortex in an anesthetized mouse (Chen et al., 2013). We extracted the histogram of the Up states duration, composed of 79 Up states that were put into bins each representing 0.0622 s (50 bins for 3.1 s). The histogram is presented in **Figure 8B**.

## 3. Results/discussion

### 3.1. The phase-space analysis unveils the origin of the peak oscillation

**Figure 1:**
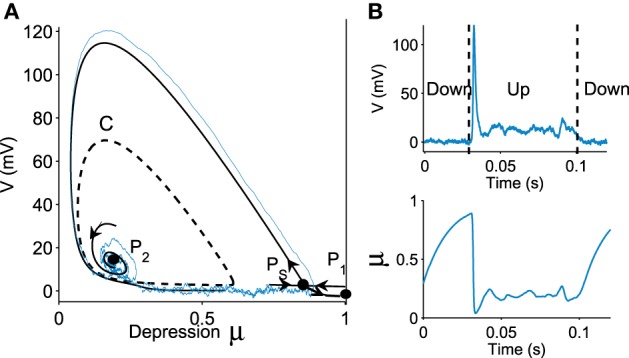
**Transition to an Up state**. **(A)** Phase portrait (*V*, μ) defined by Equation (fdt), containing a stochastic trajectory (blue). The phase space shows a limit cycle C (dashed line) containing a stable focus *P*_2_, a saddle point *P*_*S*_ and a stable attractor *P*_1_. The Up state is the domain inside the limit cycle C. **(B)** transition of a trajectory to an Up state in the time domain for the variable V and μ.

We now represent the Phase-Space (*V*, μ) in Figure 1A, which contains three critical points: two attractors *P*_1_ (located at *V* = 0, μ = 1), *P*_2_ and one saddle point *P*_*S*_. The basin of attraction for the stable focus *P*_2_ is delimited by an unstable limit cycle *C* (dashed line in Figure 1A), which defines the Up state region. This basin of attraction (Up state) appears when the network connectivity *J* exceeds a minimal value (see Holcman and Tsodyks, 2006). Indeed, as the parameter *J* increases, following a super-critical (with a minus sign) Hopf-bifurcation, the point *P*_2_, which was previously a repulser becomes an attractor and an unstable limit cycle appears around it (Figure 1A). Intuitively, this bifurcation occurs when the recurrent connections are becoming sufficiently strong.

**Figure 2:**
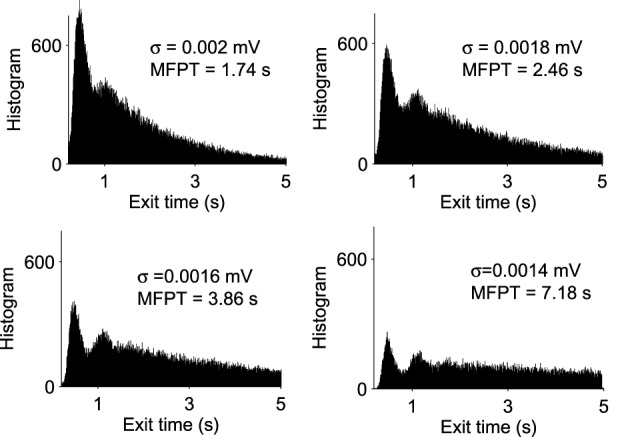
**Distribution of exit times from Up States for various noise amplitudes** (**σ** = 0.0014, 0.0016, 0.0018, and 0.002 mV). With each histogram, we computed the MFPT, which is a decreasing function of σ (total of 100,000 runs).

**Figure 3:**
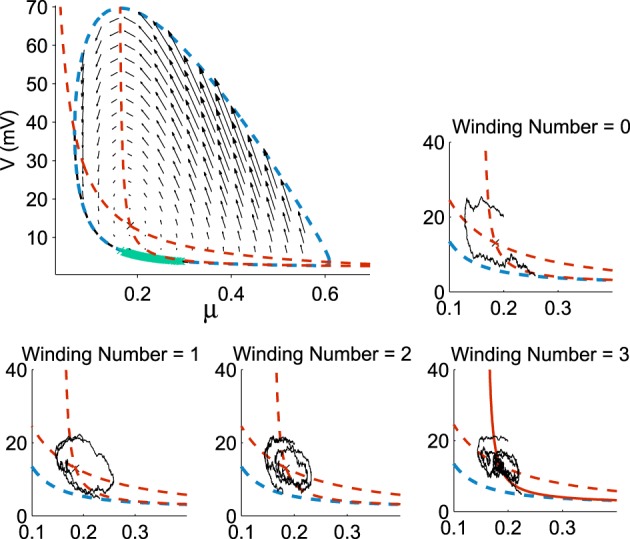
**Distribution of exit points from the Up states**. The exit points (green) for trajectories are located on a small portion of the limit cycle (dotted blue) with nullclines (dotted red) and the vector field (black arrows) (100,000 runs). In each sub-figure, a trajectory reaches the limit cycle after 0, 1, 2, and 3 rotations. All trajectories start at the initial point (μ_0_, *V*_0_) = (0.2, 20).

A trajectory enters the limit cycle by a noise induced transition (see Figure 1B). The stable focus is at the intersection of nullclines (in red in **Figure 3**). The time in the Up state is precisely the mean first passage time for a trajectory before it reaches the unstable limit cycle (dashed line) which is the basin of attraction and defines the Up state in the phase space. Once a trajectory exits through the limit cycle, the dynamics relaxes exponentially to the Down-state. We study here the distribution of times spent in the Up states (DTUS) only. The mean of this time has been estimated asymptotically and is the reciprocal of the escape rate (Schuss, 1980, 2010; Matkowsky and Schuss, 1982; Freidlin and Wentzell, 1998). It characterizes the escape in the generic activation problem. However, this analysis is not sufficient to characterize escape when the focus is located close the separatrix as it is the case here. Indeed, the DTUS shows various oscillation peaks in Figure 2, confirming that it is non-Poissonian and in addition, the amplitude of the DTUS, but not the frequency of DTUS oscillation depends on the noise size. As the noise decreases, the distribution of exit time evolves from an initially decaying exponentially to an oscillatory decay with several apparent peaks. We conclude that these peaks are a direct consequence of the dynamics induced by the geometrical organization of the recurrent ensembles, where the focus is very close to the unstable limit cycle. In the remaining part of this article, we study these peak oscillations.

**Figure 4:**
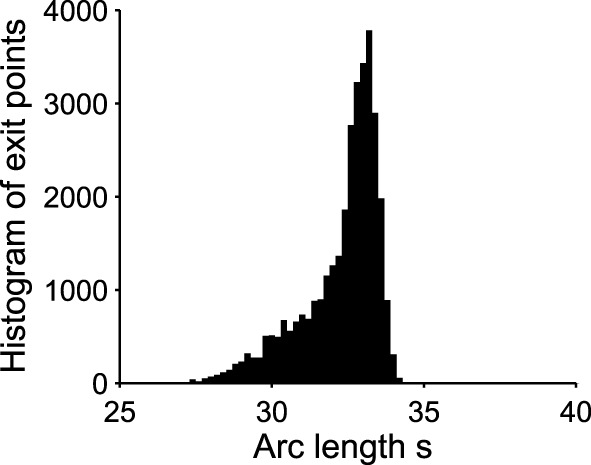
**Exit point distribution along the unstable limit cycle**. The distribution of exit points (associated to the green points in Figure 3) peaks in a very small region of the boundary. The x-axis is the arc length along the limit cycle oriented counterclockwise. The origin is (μ, *V*) = (0.035, 40) and the total arclength is 130. The initial point for all trajectories is (0.3, 30). Number of runs = 10000.

Already, we see that for a focus located near the limit cycle, the vector field vanishes close at the boundary, leading to a dynamics dominated by the noise. Thus, escape should occur in a small region of the limit cycle close to the focus. Consequently, if a trajectory does not exit in that small region, it winds around the focus before returning to the region where it can escape. This is shown in Figure 3. This rotation phenomena is responsible for the peaks in the DTUS. Moreover, the periodic peaks in DTUS is associated with a distribution of exit points concentrated in a very small part of the limit cycle, as shown in Figure 4. These periodic peaks in DTUS are in contrast with the Down-states distribution that shows a simple exponential (see Supplementary Information).

We further characterize the peak oscillations as follows: we find that the oscillation frequency in DTUS is equal to the imaginary part of the Jacobian of the deterministic vector field of system fdt at the focus point. Indeed, the coordinate of the focus in the phase-space is given by Holcman and Tsodyks (2006)

(3) $$V^{*}\;=\frac{1}{2}\left(\frac{-1+\alpha J+Ut_{r}+\sqrt{\Delta }}{\alpha Ut_{r}}\right),$$

(4) $$\mu^{*}=\frac{V^{*}}{\alpha J(V^{*}-T)}.$$

where $\Delta ={\left(1-\alpha J-Ut_{r}\right)}^{2}-4U\alpha^{2}\;Jt_{r}T$. Using parameters of Table 1, we find the coordinates (μ^*^, V^*^) = (0.1882, 12.7865). The Jacobian matrix at a point (μ, *V*) is

$$\text{Jac}(\mu ,V)=\left(\begin{array}{cc} \frac{1}{\tau }(-1+\mu J\alpha ) & -U\alpha \mu \\ \frac{J\alpha (V-T)}{\tau } & -\left(\frac{1}{t_{r}}+U\alpha (V-T)\right) \end{array}\right)$$

and the imaginary part of the eigenvalues is

(5) $${ℐ}_{\left(\mu ,V\right)}=\frac{1}{2}\sqrt{4\text{det}(\text{Jac}(\mu ,V))-{\text{Tr}}^{2}(\text{Jac}(\mu ,V))}.$$

**Figure 5:**
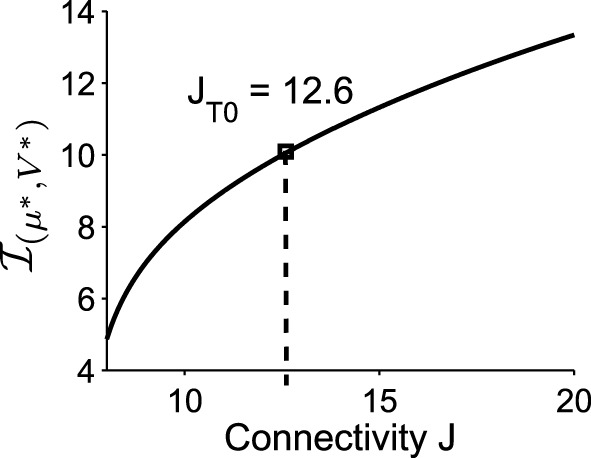
**The imaginary part of the eigenvalues of the linearized system at the Upstate**. It increases with the connectivity *J* (the value of *J* used in the previous simulations is *J* = *J*_*T*0_ = 12.6).

Applying formula Im, we find that the imaginary part of the eigenvalues of the Jacobian matrix at the Up state is ${ℐ}_{\left(\mu^{*},V^{*}\right)}=10.04.$. The associated period is thus $T_{loop}=\frac{2\pi }{{ℐ}_{\left(\mu^{*},V^{*}\right)}}=0.6258$, which is very close to the period that we obtained with Brownian simulations (see Figure 1) ${\tilde{T}}_{loop}=0.6$. This result shows that the oscillation frequency in the DTUS is determined by the deterministic property of the field at the focus only, a situation that is exact for a large class of dynamical system (Dao Duc et al., 2014). Finally, the oscillation frequency ${ℐ}_{\left(\mu^{*},V^{*}\right)}$ is an increasing function of the connectivity *J*, as shown in Figure 5.

### 3.2. Winding-decomposition for the time distribution in up state

**Figure 6:**
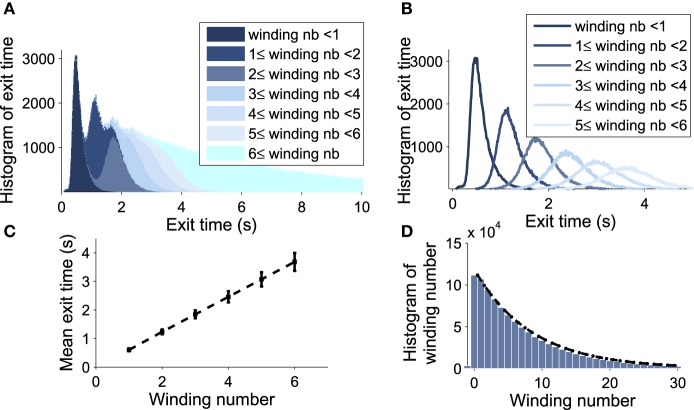
**Decomposition of the DTUS with respect to the winding number**. **(A)** Distribution of exit times. **(B)** Distribution of exit times conditioned on the winding number. **(C)** Mean exit time and variance increase linearly with the winding number. **(D)** Exponential approximation of the winding probability. The probability of escaping after *n* turns is *f*(*n*) = *p*(1−*p*)^*n*−1^, where *p* is computed empirically as the ratio of trajectories making one turn to the total number. We use 10^6^ runs, trajectories start at (μ_0_, *V*0) = (0.21, 20*mV*) with σ = 0.0015.

We explore now the contribution to the DTUS of each trajectory making exactly *k*-turns before exit. We decompose the empirical distribution of exit times by peaks, which are precisely determined by the winding number of a trajectory around the focus: the first peak is mainly composed of trajectories making no rotation, the second one is made of trajectories making exactly one rotation and so on (see Figures 6A,B). However, the dispersion of exit times for trajectories making several turns (Figure 6C) smoothes out the shape of the next peaks. To gain a better understanding of the DTUS, we decompose the exit time based on the turning number: after *k* turns, the exit time is the sum $\tau_{k}^{e}=\tau_{1}+..+\tau_{k}+\tau^{e}$, where τ^*e*^ is the time to exit without turning and τ_*q*_ is the winding time between the q*th* and *q*−1*th* turn. The variables τ_*q*_ are i.i.d because a trajectory restarts independently of its previous initial position close to the focus. Defining the probability *p* = Pr{τ^*e*^ < τ^*r*^} to rotate before exit (τ^*r*^ is the first time to complete a rotation), we use Bayes'law to decompose the distribution of exit times as

(6) $$Pr\left(\tau^{e}<t\right)=\sum_{k=0}^{\infty }Pr\left(\tau^{e}<t|k\right)Pr_{rot}\{k\},$$

where *Pr*_*rot*_{*k*} is the probability of making exactly k rotations before exit. Due to the independence of the renewal process after each rotation, it is equal to the probability to make exactly *k*−1 turns multiplied by the probability *p* to exit

(7) $$Pr_{rot}\{k\}=p{\left(1-p\right)}^{k-1}.$$

We confirm the exponential decay of the probability *Pr*_*rot*_{*k*} to exit after *k* turns (Equation W). It is well approximated by empirical stochastic simulations (Figure 6D). Furthermore, by computing the empirical ratio of the number of trajectories that exit after a single turn to the ones that rotate, we obtain the probability *p* = 0.12 (using parameters of Table 1). Finally, using the i.i.d property of the rotation times τ_*q*_, the conditional distribution probability to exit after k rotations *Pr*(τ^*e*^<*t*|*k*) is the k-convolution of the distribution of times of a single rotation *f*_1_(*t*) = *Pr*(τ_1_ = *t*) by the distribution of exit time without turning $f_{0}\left(t\right)=Pr\left(\tau^{e}=t|0\right)$:

$$Pr\left(\tau^{e}<t|k\right)=f_{0}^{*\;(k-1)}(t)\;*\;f_{0}(t)=f_{1}\;*..*\;f_{1}(t)\;*\;f_{0}(t),$$

where $f_{0}^{*\;\left(k\right)}$ is the *kth* convolution power of *f*_0_ ($f*\;g\left(x\right)=\int_{0}^{x}f\left(t\right)g\left(x-t\right)dt$). Thus, the pdf of exit time is given by

(8) $$f_{\tau }(t)=\sum_{k=0}^{+\infty }f_{1}{\;}^{*\;(k)}(t)\;*\;f_{0}(t)p{\left(1-p\right)}^{k-1}.$$

**Figure 7:**
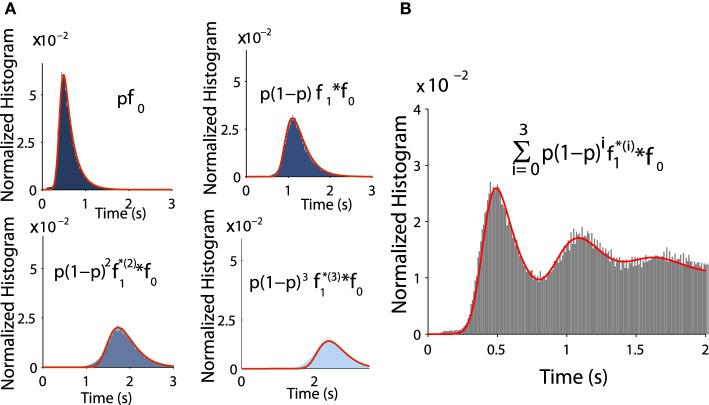
**Distribution of exit time conditioned by the winding number**. **(A)** Comparison between histograms of trajectories of 1, 2, 3, and 4 turns and formulae f0 and fk (in red) **(B)** Comparison between the histogram of exit time obtained from subfigure A with the analytical formula (in red) Equation (8).

To validate Equation (tau), we estimated $f_{1}^{*\;\left(k\right)}\left(t\right)\;*\;f_{0}\left(t\right)$ for all successive peaks and compared them to Brownian simulations as follows: the distributions *f*_0_ and *f*_1_ are exponentially distributed at infinity and can be approximated by

(9) $$f_{k}(x)=C_{k}\left[1+er\;f\left(\frac{x-a_{k}}{b_{k}}\right)\right]e^{-\lambda_{k}x},$$

where *k* = 0, 1 and the normalization constants *C*_*k*_ are obtained by $\int_{0}^{+\infty }f_{k}\left(t\right)dt=1$. Fitting the empirical histograms conditioned on a winding number < 1, we obtain for *f*_0_ the following parameters *a*_0_ = 0.43, *b*_0_ = 0.1, λ_0_ = 4.8. By simulating trajectories that are making one turn, we obtain for *f*_1_: *a*_1_ = 0.48, *b*_1_ = 0.1, λ_1_ = 5.5. Then we use these analytical expressions $f_{0}^{*\;\left(k\right)}\left(t\right)p{\left(1-p\right)}^{k-1}$ and compare them with the Brownian simulations (see histograms obtained for *k* = 1, 2, 3, 4 in Figures 7A,B).

The oscillations in the Up state histogram shown in Figure 7 were not reported before and are for instance completely different from the stochastic amplification phenomena for the voltage in the Up states reported in Hidalgo et al. (2012), which concerns fluctuations of the mean firing rate with a peak in the power spectrum. This effect is fundamentally different: while the frequency of the peak depends on the noise amplitude in Hidalgo et al. (2012), it is independent here and determined only by the Jacobian of the deterministic system at the attractor.

Moreover, the oscillation peaks that we found here are a consequence of the close proximity between the two-dimensional limit cycle and the point attractor. For that same reason, our findings also differ from that of Mejias et al. (2010), in which the stochastic dynamical system is reduced to a one dimensional Ornstein-Uhlenbeck process. Computing the power distribution under this reduction implies that the attractor is far enough away from the basin of attraction of the Up state. However, this situation is not the one obtained from parameters accounting for physiological conditions (Tsodyks and Markram, 1997; Holcman and Tsodyks, 2006; Dao Duc et al., 2015), where the attractor is located in the boundary of the basin of attraction (see Figure 1). In this case, the exit time distribution cannot be well characterized simply by the first eigenvalue of the associated Fokker-Planck equation. Indeed, the second one is also needed and contains an imaginary component (Dao Duc et al., 2014), a phenomenon that is only possible for nonself-adjoint operator in two dimensions at least.

### 3.3. Extracting the neuronal network connectivity from the distribution of times in up states

The distribution of times in Up states is non-Poissonian, because the underlying dynamics is a non-classical escape problem (Holcman and Schuss, 2014). Analysis of the Fokker-Planck equation for generic dynamical systems (Dao Duc et al., 2014) reveals that the distribution of time can be expanded in eigenvalue series

(10) $$f_{Up}(t)=A_{0}e^{-\lambda_{0}t}+\sum_{n,m}C_{n,m}e^{-\lambda_{n,m}t},$$

where *A*_0_, *C*_*m, n*_ are constants, λ_*n, m*_ complex-valued higher-order eigenvalues and λ_0_ is the principal eigenvalue of the Fokker-Planck in the domain that defines the Up state (basin of attraction delimited by an unstable limit cycle). The second order approximation is

(11) $$f_{Up}(t)=Ae^{-\lambda_{0}t}+Be^{-\lambda_{1}t}\cos(\omega t+\phi )$$

where *A, B* are constants and the intrinsic parameters are λ_0_, λ_1_, ω, ϕ. All these constants needed to be identified. The theory predicts (Schuss, 2010) that $\lambda_{0}\approx \frac{1}{\bar{\tau }}$, which is the mean time the voltage membrane stays in Up states. It can be directly estimated from the histogram by computing the mean (first moment). The other parameters should be fitted from the histogram with the constraint that λ_1_ > λ_0_ and $T=\frac{2\pi }{\omega }$ is the period between two consecutive peaks and can be determined from histograms.

**Figure 8:**
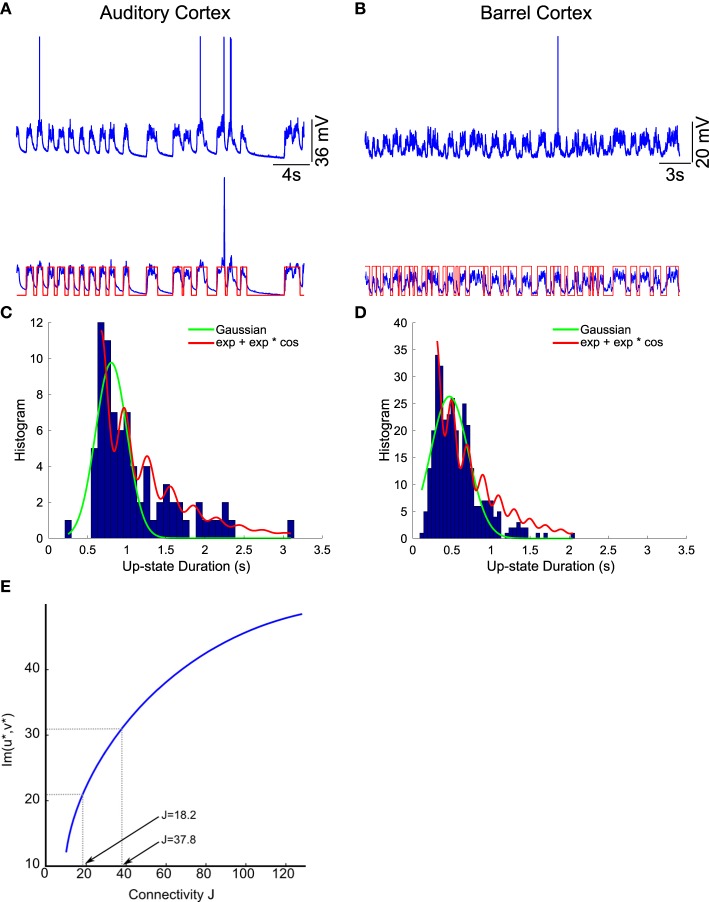
**Two electrophysiological recordings (top) and their annotation after processing (bottom)**. **(A)** An entire 32 s recording from a L2 cortical neuron of the auditory cortex. **(B)** A 30 s sample of a recording from a L4 cortical neuron (barrel cortex). Histogram of Up states duration obtained from intracellular *in vivo* recordings from neurons in auditory **(C)** (with error *R*^2^ = 0.73 Gaussian and *R*^2^ = 0.89 for the model *f*_*Up*_) and barrel cortex **(D)** (with error *R*^2^ = 0.85 Gaussian and *R*^2^ = 0.90 for the model *f*_*Up*_). We fitted each distribution with a Gaussian curve (green) and the function *f*_*Up*_(*t*) = $Ae^{-\lambda_{0}t}+Be^{-\lambda_{1}t}\cos\left(\omega t+\phi \right)$ (red), predicted by our model. The parameters of the fit are summarized in Table 2. The fit reveals the peak oscillations predicted by the depression mean-field model. **(E)** Imaginary part or period of the peaks as a function of the connectivity *J* (τ = 0.02). We obtained the connectivity *J* = 18.2 and 37.8.

We apply the present theory to two different intracellular recordings obtained in the barrel cortex and the auditory cortex (see Section 2) (Figure 8) and we obtain for the first exponent λ_0_ = 1.92 and λ_0_ = 1.48 respectively (see Table 2). We found that the period interval between two consecutive peaks $T=\frac{2\pi }{31}=0.2$ and $T=\frac{2\pi }{21}=0.29$, leading respectively to *J* = 37 and *J* = 18.2, where we used the graph presented in Figure 8. We can recover the connectivity from the imaginary part of the eigenvalue of the field at the attractor, which is exactly the period *T*. We obtained for each case the degree of connectivity using τ = 0.02. The fitted curves are represented in Figure 8 where we also plotted Gaussian fits $f\left(t\right)=A\exp\frac{-{\left(t-B\right)}^{2}}{C}$ (Table 3). It is remarkable that in both cases, the first and the second eigenvalues λ_0_ and λ_1_ are sufficiently close, leading to the oscillation phenomena in the distribution of exit times.

**Table 2:** **Fitting parameters**.

**Recordings**	**A**	**B**	**λ_0_**	**λ_1_**	**ω**	**ϕ**
					**(Frequency)**	**(Phase)**
Barrel cortex	54.4438	13.100	1.92	1.99	31.41	−3
Auditory cortex	10	1.48	1.87	21.22	23.9135	−4.3

**Table 3:** **Gaussian fits**.

**Recordings**	**A**	**B**	**C**
Barrel cortex	14.96	0.4703	0.3448
Auditory cortex	6.582	0.8091	0.2978

We conclude that in both cases, we obtain that the distance between two peaks is the period *T* = 0.2, which is related to the imaginary of the eigenvalue at the attractor. Using Figure 8, we obtain that the degree of connectivity *J* responsible for such dynamics is *J* = 30. It is possible to relate the abstract degree of connectivity *J* used in the equations to the actual number of synapses by calibrating the model with experimental data. This procedure has been applied in neuronal cultures, where we recently related the connectivity *J* = 40 (for τ = 0.05) to an approximating 3000 synapses per neuron in hippocampal islands containing 5–30 neurons homogenously connected (Dao Duc et al., 2015). Finally, the present analysis based on the depression-facilitation model differs significantly from the hidden-Markov model approach (McFarland et al., 2011), where several exponential distributions including the gamma distribution and inverse Gaussian distribution and the geometric distribution were used to fit the distribution.

## 4. Conclusion

Using a mean-field model, we have shown here that the distribution of times in the Up state of the neuronal membrane potential shows several oscillation peaks. These oscillations are due to the intrinsic synaptic dynamics in the depression model. The peaks frequency is independent of the noise amplitude, but depends on the amount of synaptic connection (Figure 5). It is conceivable that neurons have found a possibility to exploit this quantification property. A possible prediction of the model is that working memory could also show period time, where the histogram of durations show multiple peaks, a phenomena that would be interesting to demonstrate experimentally. The peak oscillation phenomena described here is a generic effect of stochastic dynamical systems where recurrent set (focus point here) are located close enough to the boundary.

Another aspect of the present work is the possibility to extract the mean network connectivity from the distribution of peaks, located in the Up state time series histogram. This analysis is a direct consequence of the model, from which we show the distance between two consecutive peaks depends on network connectivity (mean number of synapses per neurons). In future work, more general models, including inhibition, intrinsic electrical properties of neurons, should be used to recover more information about the network connectivity from the distribution of times of the mean neuronal membrane voltage or in general from the distribution of firing rates, which could be applied to neuronal network of various sizes.

### Conflict of interest statement

The authors declare that the research was conducted in the absence of any commercial or financial relationships that could be construed as a potential conflict of interest.
