# Supplementary material for: Synaptic dynamics and neuronal network connectivity are reflected in the distribution of times in Up states
Source: Front Comput Neurosci. 2015 Jul 29;9:96. doi: 10.3389/fncom.2015.00096 (PMC4518200; doi:10.3389/fncom.2015.00096)
Supplement: Supplementary file 1 [file Presentation1.PDF]

Supplementary Information Neuronal Network Connectivity and the Distribution of Times in Up-States

K. Dao Duc[1] P. Parutto[1] X. Chen[2] J. Epsztein[3] A. Konnerth[2] D. Holcman[1] *

March 6, 2015

In this supplementary information, we show that the oscillation peaks in the distribution of time in the Up-states is also present in a mean-field model with inhibition. We further show that there are no oscillation peaks in the down-state distribution and the decay follows a single exponential.

1 Up-Down state mean-field model with Inhibition

We show here that adding inhibition does not affect the oscillation property for the time spent in the Up-state. We use the classical mean-field model of [1], where added an inhibitory component that can also be driven by

*[1]Ecole Normale Supérieure, 46 rue d'Ulm 75005 Paris, France. [2] Institute of Neuroscience Biedersteiner Str. 29, D-80802 Munchen Germany. [3] Institute of Neurobiology INMED-INSERM U901 Marseille, France

depression, formulated as

$$\begin{cases} \tau\dot{V}_E &= -V_E + J_E\mu_E U R(V_E) - \mu_I U J_{IE} R(V_I) + \sqrt{\tau}\sigma\dot{\omega}_1 \\ \tau\dot{V}_I &= -V_I - J_I\mu_I U R(V_I) + \mu_E U J_{EI} R(V_E) + \sqrt{\tau}\sigma\dot{\omega}_2 \\ \dot{\mu}_E &= \frac{1-\mu_E}{t_r} - U\mu_E R(V_E) \\ \dot{\mu}_I &= \frac{1-\mu_I}{t_r} - U\mu_I R(V_I), \end{cases} \tag{1}$$

where V_E, μ_E (resp. V_I, μ_I) are the average voltage (in mV) and depression variables of the excitatory (resp. inhibitory) population. J_E and J_I are the average synaptic strength of the two populations while J_{EI} ($E \to I$) and J_{IE} ($I \to E$) represent the average synaptic strength of the connections between the Excitatory and Inhibitory populations. ω_1, ω_2 are two independent δ-correlated white noises of mean zero and variance one. All other parameters are given in table 1.

Parameters	J_E	J_I	J_{EI}	J_{IE}
Values	0.0156	J_E	$2J_E$	$0.2J_E$

Table 1: Parameters for the two-population model

We study the dynamics in three different cases: first, the parameters are given in table 1 (fig. 1A-B), we then study the case where there is no feedback on the inhibition. the parameters are $J_{EE} = 0.0195$; $J_{IE} = 0.2 * J_{EE}$; $J_{EI} = 2 * J_{EE}$; $J_{II} = 0$. Finally we increase the inhibition feedback to $JEE = 0.024$; $J_{IE} = 0.4 * J_{EE}$; $J_{EI} = 2 * J_{EE}$; $J_{II} = J_{EE}$. In all cases, the Up-Down state behavior can be observed as show in in Fig. 1A-F. In the main of the manuscript, we study the excitation only.

2 Distribution of times in the Down-state

Using the mean-field equations with no inhibition (see main text), we obtained statistics for the time in the Down-state ($V \leq 2.37$, which is voltage-position of the saddle point in the phase-space). We find that the distribution

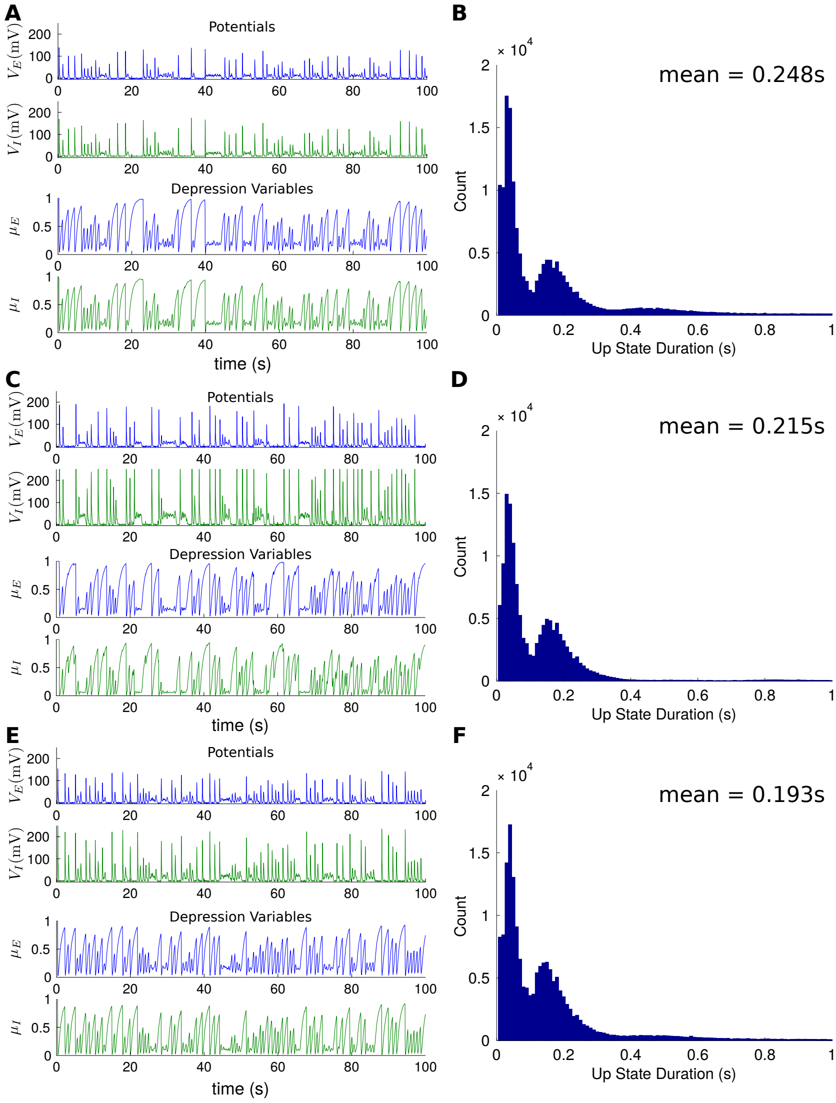

Figure 1: **Up-Down state mean-field model with Inhibition. A.** Dynamics of the 4 parameters: V_E, VI, μ_E, μ_I**B.** histogram of the time in Up and down states. **C-D.** like in **A.** with $J_{EE} = 0.0195; J_{IE} = 0.2 * J_{EE}; J_{EI} = 2 * J_{EE}; J_{II} = 0$. **E-F.** parameters are $JEE = 0.024; J_{IE} = 0.4 * J_{EE}; J_{EI} = 2 * J_{EE}; J_{II} = J_{EE}$.

Parameters	a (95% conf. int.)	b (95% conf. int.)	R^2
Simulations	2188 (2183, 2194)	-2.38 (-2.39, -2.37)	0.99
Auditory Cortex	30.77 (19.05, 42.5)	-1.82 (-2.76, -0.88)	0.77
Barrel Cortex	192.9 (163.5, 222.3)	-10.72 (-12.42, -9.02)	0.98

Table 2: Fitted parameter and error for the Down-state duration.

is well approximated by single exponential exponential (Fig. 2a), in agreement with the classical Kramer's escape problem from an attractor. We further estimated the distribution of times in the Down-state from for experimental data (Fig. 2b-c). We also found that the distribution is well approximated a single exponential, in agreement with the predictions of the model, as shown in fig. 2 and table 2. We fitted the data with a single exponential model $f(f) = ae^{bt}$, using the fitting toolbox of `Matlab`. For the Auditory and Barrel Cortex recordings, we set downs states as regions for which the membrane potential is less than 6mV above the resting potential. In order to keep the baseline constant we subtracted to the data the moving average with a window of 10s (30s for Barrel cortex data) we then subtracted the minimal value of the data to have only positive values.

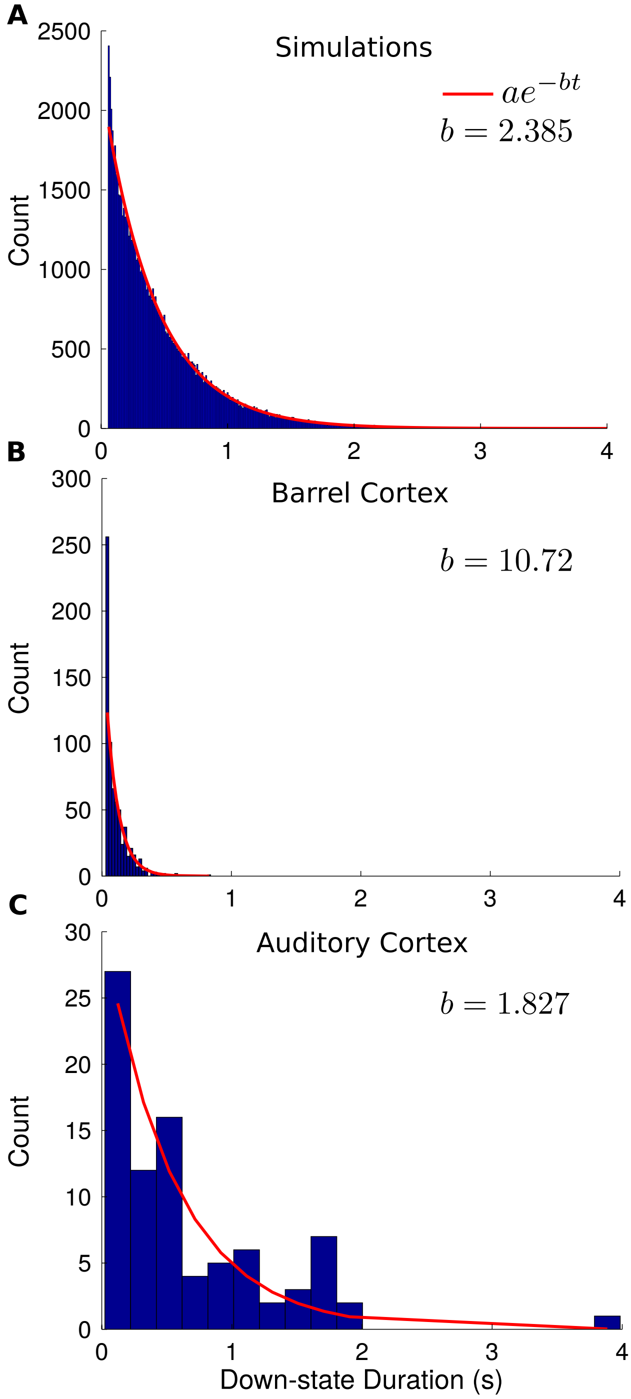

Figure 2: **Distribution of time in the Down-state.**

References

[1] Holcman, D.Tsodyks M. (2006) The Emergence of Up and Down States in Cortical Networks. *PloS Computational Biology*, 3,2
